# Procedure for spotted fever group *Rickettsia* isolation from limited clinical blood specimens

**DOI:** 10.1371/journal.pntd.0010781

**Published:** 2022-10-14

**Authors:** Marah E. Condit, Emma Jones, Brad J. Biggerstaff, Cecilia Y. Kato

**Affiliations:** 1 Rickettsial Zoonoses Branch, Division of Vector-Borne Diseases, National Center for Emerging and Zoonotic Infectious Diseases, Centers for Disease Control and Prevention, Atlanta, Georgia, United States of America; 2 Office of the Director, Division of Vector-Borne Diseases, National Center for Emerging and Zoonotic Infectious Diseases, Centers for Disease Control and Prevention, Fort Collins, Colorado, United States of America; Mahidol Oxford Tropical Medicine Research Unit, THAILAND

## Abstract

**Background:**

Current isolation techniques for spotted fever group *Rickettsia* from clinical samples are laborious and are limited to tissue, blood and blood derivatives with volumes ideally greater than 1 mL. We validated the use of simplified methodologies for spotted fever group *Rickettsia* culture isolation that overcome sample volume limitations and provide utility in clinical diagnostics and research studies.

**Methodology/Principal findings:**

A modified cell culture method is evaluated for the isolation of *Rickettsia* ssp. from human diagnostic samples. Culture sampling method, culture platform, and growth phase analysis were evaluated to determine best practices for optimal culture isolation conditions. Rickettsial isolates (*R*. *conorii*, *R*. *rickettsii*, and *R*. *parkeri*) were grown in Vero E6 cells over a course of 5 to 7 days at low inoculum treatments (~40 bacterial copies) to standardize the sampling strategy at a copy number reflective of the bacteremia in acute diagnostic samples. This methodology was verified using small volumes (50 μL) of 25 unprocessed clinical whole blood, plasma, and serum samples from acute samples of patients suspected of having Rocky Mountain Spotted Fever, of which 10 were previously confirmed positive via the PanR8 qPCR assay, 13 had no detectable *Rickettsia* DNA by the PanR8 qPCR assay, and 2 were not previously tested; these samples resulted in the cultivation of 7 new *R*. *rickettsii* isolates.

**Conclusions/Significance:**

We observed that rickettsial isolate growth in culture is reproducibly identified by real-time PCR testing of culture media within 72 hours after inoculation. Additionally, specimen sedimentation prior to isolation to remove red blood cells was found to decrease the amount of total organism available in the inoculum. A small volume culture method was established focusing on comparative qPCR detection rather than bacterial visualization, taking significantly shorter time to detect, and requiring less manipulation compared to traditional clinical isolate culture methods.

## Author summary

Spotted fever group *Rickettsia* (SFGR) are Gram-negative intracellular bacteria that during early onset localize in blood vessel endothelium before disseminating in the bloodstream and lymph via lymphocytes and other non-endothelial cells. Morbidity and mortality vary by species, with fatality rates up to 37% of PCR-positive cases in Mexico (*Rickettsia rickettsii)*, with outcomes dependent on patient access to healthcare. SFGR disproportionately affects those in impoverished communities worldwide, and presentation is often with nonspecific symptoms, making early diagnosis difficult. Advances in acute laboratory diagnostics are critical to improve timely confirmatory diagnosis and our understanding of disease progression. Clinical culture and isolation methodologies for SFGR are largely unchanged since the development of the shell vial technique in 1989. We validated an isolation method that utilizes small volumes of acute clinical sample and minimal culture manipulation by first determining best practices for sample preparation, sampling methodology, and timing of sampling. This optimization resulted in detection of low copy number inoculums as early as 2 days in both cellular monolayer and culture supernatant samples. Initial testing was done across multiple SFGRs to demonstrate that species with varying virulence can be isolated using similar parameters. This simplified, efficient method for SFGR isolation from clinical samples has the potential to compliment and improve diagnostic testing, reflex testing, isolate characterization, and research studies.

## Introduction

Laboratory diagnostics of rickettsial infections at the acute stage of illness has many challenges due to the low level of circulating bacteria in blood and a lack of a reliable and consistent antibody response at this stage of illness [1–3]. Reported bacteremia ranges from 10^6^ copies per mL in fatal cases, to fewer than 100 bacterial copies per mL in the peripheral blood of patients in the early acute phase of Rocky Mountain Spotted Fever (RMSF) illness [2,3]. *Rickettsia* are Gram-negative obligate intracellular bacteria that localize in the vascular endothelium and disseminate through the body via the bloodstream and lymph likely via lymphocytes, and other non-endothelial cells [4,5]. *In vivo* and *in vitro* analyses of virulence suggest that the level of circulating bacteria varies by species and strain of spotted fever group *Rickettsia* (SFGR) [6–8]. Cultivation and isolation of the illness causing bacteria enhances molecular diagnosis and allows for further characterization. Molecular detection begins with appropriate blood or blood derivative sample collection at the early stage of illness, while the patient is symptomatic, and before or within 48 hr of doxycycline administration. Continuous storage at 2–8°C, prompt transport, and nucleic acid extraction within 7 days of sample collection is also necessary for maintaining specimen integrity. Sensitive qPCR detection, such as with the PanR8 Pan-*Rickettsia* assay, which has a limit of detection of approximately 1,800 genomic copies per mL (~9 copies per reaction, with 95% efficiency), is another critical component to achieve an accurate diagnosis [9]. While diagnostic tests are being performed, multiple sample nucleic acid extractions may be required depending on the disease differential requested for testing, increasing the potential for specimen depletion. For culture isolation, most published protocols utilize at least 1 mL of sample per attempt [10,11] to overcome the low bacterial loads and specimen processing methods, and specimen collection must be before or at the time of doxycycline treatment for these sample types.

While the different etiologic agents of SFGR may present clinically with similar signs and symptoms, including fever, malaise, headache, and maculopapular rash [12,13], each species has different growth dynamics and virulence [14]. Differences in virulence may be observed as differences in disease outcome and severity [8,15–20]. Infection with *R*. *rickettsii* (Rri), the etiologic agent of RMSF, has an estimated fatality rate of 5–10% in the U.S. [13] and up to 37% in Mexicali, Mexico [20]. *R*. *conorii* (Rco), the etiologic agent of Mediterranean Spotted Fever, has a fatality rate of up to 32.3% [21]. *R*. *parkeri* (Rpa) infection (*R*. *parkeri* rickettsiosis) results in mild symptoms, is associated with no known deaths [13], and is thought to be commonly misdiagnosed [12].

Cultivation of *Rickettsia* from clinical specimens is performed to augment diagnosis in some reference laboratories. The use of shell vial culture method was adapted for rickettsial culture from a cytomegalovirus assay in 1989 [10] and is used in clinical reference laboratories worldwide with varying efficiencies [10,11,22–28]. In brief, whole blood (WB) samples are sedimented to concentrate the *Rickettsia* and remove red blood cells (RBC), which cause background interference during the stain evaluation [29] and disrupt cell monolayers. Early work isolating *R*. *rickettsii* from infected guinea pig primary blood monocytes in shell vials [30] included RBC removal, and this has remained standard practice. Resulting plasma and buffy coat (BC) layers are inoculated into 3–4 shell vials traditionally containing HEL or MRC5 cells grown on coverslips inserted into the shell vials. The shell vials are then centrifuged at low speed to enhance the rickettsial attachment and penetration of cells [22,25,31], after which the clinical material is removed and replaced with fresh media [11], which is changed regularly (every 2–3 days) [10,30], and incubated for 3–15 days. Once growth is observed, the culture is monitored for three passages prior to preforming PCR screening [22].

We established a simplified small volume culture model using 10 cm^2^ culture tubes with limited culture manipulation, using SFGR species of varying virulence: Rco Malish 7, Rpa Coweta, and Rri AZ3. Evaluation of the model was done using low copy number inocula of Rco, Rpa, and Rri to mimic acute clinical samples. Real-time PCR detection was observed before the establishment of visible cytopathic effects (CPE) of the monolayer as early as 72 hours after inoculation, consistent with what is described for shell vial isolation [10]. Validation was done with a total of 25 acute clinical blood, serum, and plasma samples: 16 drawn before or at the time of doxycycline administration, 6 initially tested positive via the PanR8 assay [3]; 7 drawn after doxycycline administration, 2 initially tested positive via the PanR8 assay; and 2 with unknown doxycycline administration status, both initially positive via the PanR8 assay. This clinical sample validation confirmed data on appropriate sample parameters required to increase the likelihood of a successful SFGR isolation [22,32] and demonstrates successful isolations with the minimal manipulation small volume model.

## Materials and methods

### Ethics statement

The Emory University Institutional Review Board approved IRB protocol number IRB00045947 to collect ethylenediaminetetraacetic acid (EDTA) whole blood with formal written patient consent. The Centers for Disease Control and Prevention (CDC) Institutional Review Board approved protocol 7014 to de-identify routine diagnostic specimens to be used for secondary research purposes only and no further review was required for this study. No patient consent was obtained as the data were analyzed anonymously as per the protocol.

### Rickettsial strains and semi-pure rickettsal inocula preparation

*R*. *conorii* Malish 7 strain [33], Rri AZ3 strain [17,34] and Rpa Coweta strain were obtained from the CDC Rickettsial Isolate Reference Collection (CRIRC), Rickettsial Zoonoses Branch, Centers for Disease Control and Prevention, Atlanta, GA. Rco was cultured at passage 4 to 5; the passage number prior to obtaining this isolate is unknown. Rri was isolated in 2004 and used at passage 7 to 8, and Rpa was isolated in 2014 and used at passage 3 to 4. All cultures were grown in Vero E6 cells in Eagle’s Minimum Essential Media (EMEM) (VWR, catalog# 12-125F) supplemented with 5% FBS (Atlanta biologicals, catalog# S12650), 0.1 mM NEAA (Gibco, Catalog# 13-114E), 10 mM HEPES (Gibco, catalog# 15630080), 2 mM L-glutamine (Gibco, catalog# 25030–081), 10 mM sodium pyruvate (Lonza, catalog# 13-115E) (5% EMEM) in a humidified incubator with 5% CO_2_ at 34°C [35]. Infected 75 cm^2^ flasks were incubated until ≥ 50% CPE was observed (Rri, Rco) or was 90–100% infected with no CPE (Rpa), determined by acridine orange staining (BD, catalog# 212536) [36]. At this time, monolayers were dispersed by sterile glass beads (RCO and RPA) or just supernatant was taken (RRI) and centrifuged at 500 × g for 3 minutes at 4°C to remove the majority of Vero E6 cellular debris, followed by supernatant centrifugation at 17,000 × g for 30 minutes at 4°C. The supernatant was discarded and the resulting rickettsial bacterial cell pellets were suspended in 10 mL of sucrose phosphate glutamate buffer (SPG) [37]. Single use semi-pure rickettsial inocula preparations of 50 μL aliquots were created by diluting the prep 1:100 in SPG and quantified by the PanR8 qPCR assay and stored at -80°C.

### Cell culture and infection

Vero E6 cells were cultured as above in 10% FBS supplemented media. Cells were seeded at ~4x10^6^ cells (25 cm^2^ flask, Corning, catalog # 430639) and ~8x10^5^ cells (10 cm^2^ culture tube, Techno Plastic Products, catalog# 91243) 24–48 hours before inoculation, at 37°C with 5% CO_2_, and inoculated at 95–99% cell confluence. Inocula were standardized to ~40 copies added to 6 mL or 3 mL of 5% EMEM, for 25 cm^2^ flasks and 10 cm^2^ culture tubes, respectively. Cultures were incubated for 5–7 days in a humidified incubator at 34°C with 5% CO_2_. No media changes occurred. dx.doi.org/10.17504/protocols.io.bxbmpik6

### Time course determination

**Fig 1:**
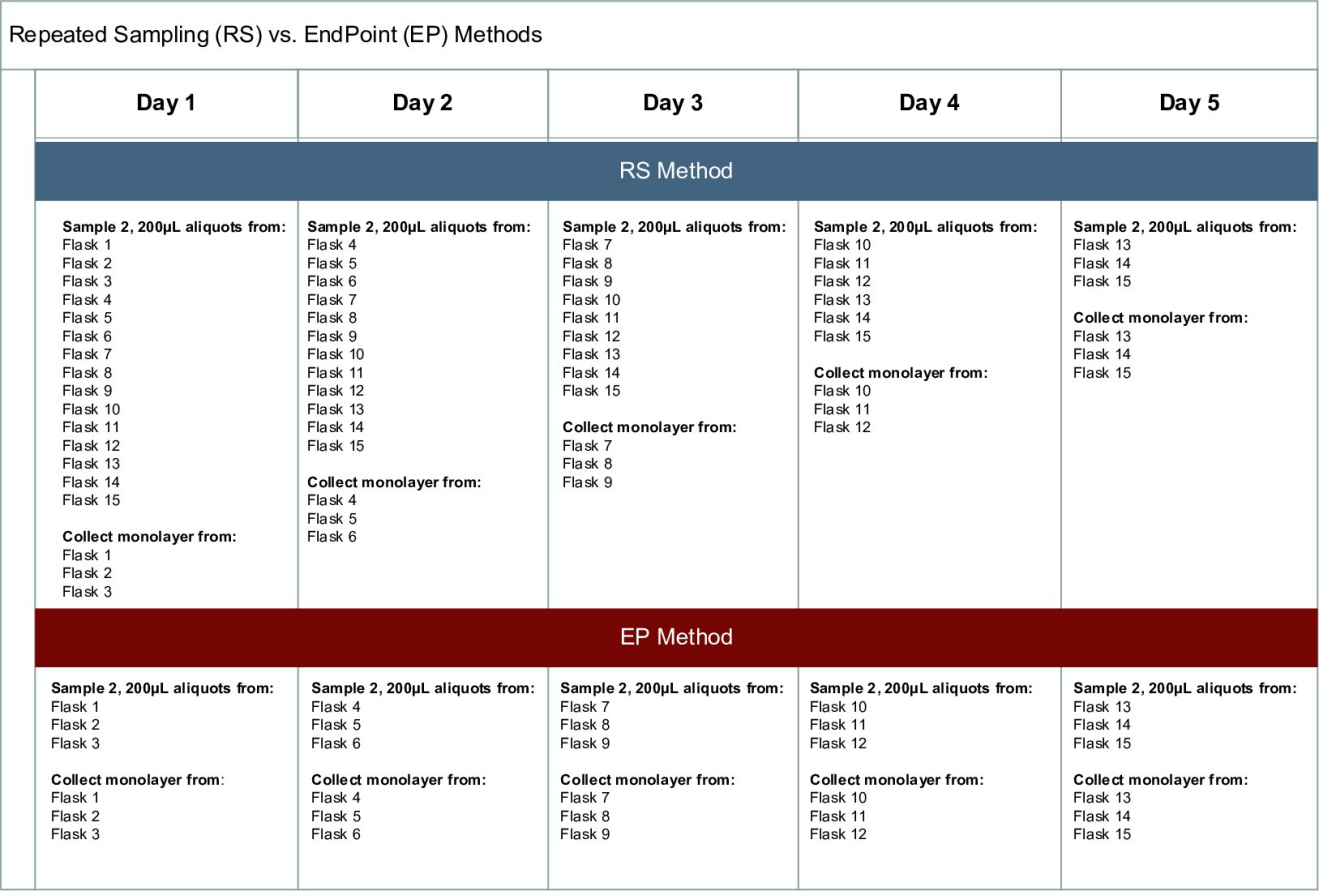
Repeated Sampling (RS) vs. Endpoint Method Plan.

Sampling was employed using two methods. For the repeated-sampling method (RS), duplicate 200 μL supernatant samples from 15 (25 cm^2^) flasks were sampled daily for 5 days with 3 flasks’ monolayers collected daily. This method decreases a flask’s volume by 400 μL at each sampling and the number of flasks by 3, daily. For the endpoint (EP) method, 15 culture apparatuses were used with duplicate 200 μL supernatant samples from three 25 cm^2^ flasks or three 10 cm^2^ culture tubes were sampled followed by monolayer collection daily, for the maintenance of a constant volume over time, Fig 1. Data from Rco experiments sampled on days 1–5 determined timing of sampling for Rpa and Rri experiments on days 3–7. Daily monitoring for CPE was performed with a Zeiss Vert.1A light microscope. Collected cells had monolayers washed with 1X HBSS (Gibco, catalog # 14175–095), followed by the addition of 0.05% Trypsin EDTA (Gibco, catalog# 25300054). Flasks were incubated at 34°C with 5% CO_2_ for 10 minutes or until cells lifted by tapping. Trypsin was quenched with 5% EMEM and centrifuged at 17,000 × g for 30 min at 4°C. Cell pellets were suspended in 1X PBS, (Gibco, catalog# 10010–031) for a total volume of 800 μL, then 200 μL of which was sampled in duplicate for DNA extraction.

### Viability determination

Duplicate 50 μL supernatant samples were taken from each of the three 25 cm^2^ flasks at the time of cell collection (EP study). Samples were inoculated into a 24 well plate containing 99% confluent Vero E6 cells with 5% EMEM and incubated 4–13 days until CPE was observed [19]. Growth was confirmed by acridine orange stain (BD, catalog# 212536) on cytospin prepared slides of monolayer scrapes [36].

### Blood acquisition

Whole blood (WB) from 1 healthy donor was obtained through the Emory University Centers for Transfusion and Cellular Therapies and the CDC Serum Bank. Ethylenediaminetetraacetic acid (EDTA) whole blood was collected with written patient consent under approved IRB protocol number IRB00045947, stored at room temperature (RT), and processed within 2 hours from the time of draw. The donor was screened and confirmed negative for HIV-1/HIV-2 antibody, Hepatitis C antibody, Hepatitis B (Surface antigen and Core antibody), NAT Triplex (HIZ-a/HCV/HBV), HTLV I/II antibody, Syphilis, West Nile virus (RNA) and *T*. *cruzi* prior to blood draw. Blood was confirmed for the absence of SFGR via the PanR8 qPCR assay.

### Blood spiking, fractionation, and assessment

Five mL of EDTA whole blood were inoculated with 1,800 copies/mL of Rco or Rri, incubated with gentle rocking at RT for 30 min, and aliquoted into 1 mL volumes. Individual aliquots were fractionated by overlaying over 5 mL of 1.077g/mL polysucrose and sodium diatrizoate, Histopaque 1077 (Sigma, catalog# 10771). The inoculated blood was then centrifuged at 800 × g for 20 min at RT with high acceleration and low brake, per manufacturer instructions. Total plasma, BC and RBC layers were analyzed by qPCR for DNA quantitation, as described below. Extractions were performed on the same day as the fractionation from 200 μL aliquots, except from the Rri RBC layer, which was 100 μL with an equal volume 1X PBS.

### Diagnostic sample evaluation

Clinical specimens received in the Rickettsial Diagnostic Laboratory, CDC (Atlanta, GA) in 2018–2019 for routine diagnostics were de-identified as per IRB protocol 7014. Validation was done with a total of 25 acute clinical blood, serum, and plasma specimens which met the minimal sample criteria: acute samples of appropriate sample type (WB, serum, plasma) and having SFGR molecular analysis as the original test request. Of these 16 were drawn before or at the time of doxycycline administration, 6 initially tested positive via the PanR8 assay [3]; 7 drawn after doxycycline administration, 2 initially tested positive via the PanR8 assay; and 2 with unknown doxycycline administration status, both initially positive via the PanR8 assay. Fifty microliters of each specimen were combined with 50 μL of SPG then frozen at -80°C in a CoolCell cell freezing container (Biocision) until the time of isolation or 50 μL of fresh sample were combined with 50 μL of SPG and inoculated at the time of receipt in the lab (specimens C016, C017). The total volume was inoculated directly into 10 cm^2^ culture tubes containing 3 mL 5% EMEM, incubated at 34°C with 5% CO_2,_ and specimens C001-C017 were sampled as described above for up to 11 days, with monolayers frozen at -80°C in SPG. Specimen C001 had confirmed growth by day 7. Specimen cultures C002-C017 were thawed for verification of isolation and passaged in 25 cm^2^ culture flasks in 5% EMEM, grown for up to 18 days, or until visual growth was noted. Specimens C018-C029 were sampled for 11 days and monitored for up to day 22 in culture post-inoculation with the addition of 2 mL 5% EMEM to overcome volume loss on day 11.

### Quantification by quantitative real-time PCR

Total nucleic acid extraction was performed using the MagNA Pure Compact Nucleic Acid Isolation Kit 1 (Roche, Catalog# 03730964001) with a 200 μL elution volume, as per manufacturer’s guidelines for external lysis. All samples were heat inactivated in lysis buffer consisting of 280 μL MagNa Pure LC Total Nucleic Acid Isolation Kit (Roche, catalog# 03246779001) with 20 μL Proteinase K (Roche, catalog# 03115828001) for 30 min at 56°C before extraction. Five μL of extracts were quantified by the PanR8 qPCR assay [9] with standard curve of positive control plasmid from 10,000 copies to 0.1 copies. All samples and standard curves were run in duplicate on a 7500 Fast Dx Real-Time PCR System (Applied Biosystems), and each plate included a standard curve, positive control, and no template controls.

### Data analysis

All qPCR results are expressed in total copies per culture apparatus as determined by multiplying the calculated copy number per μL by both the total volume of the flask at the time of sampling and amplification values based on standard curves with R^2^ values of 0.97–0.99. Doubling times and inocula calculations are expressed as mean ± standard deviation. Coefficient of variation (CV) is used to express variability in replicate values relative to the mean. R version 4.0.3 (R Foundation for Statistical Computing, Vienna, Austria) was used to analyze differences in log_10_-transformed copy values between study designs across days and within sample type. A linear model, fitted using generalized least squares, was used to account for unequal variances across time points and between study designs. Accumulation curves were compared by study design using a time by study design interaction parameter. Summary statistics of culture growth at each timepoint were calculated as geometric means (GM) rather than arithmetic means due to growth data being skewed. Transforming growth data onto the log_10_ scale allows for the means and confidence intervals to be calculated on data that are more normally distributed and therefore better approximate assumptions of normality made when calculating means and confidence intervals, before transforming back to the original scale for interpretation. Upper confidence limits were replaced with infinity (Inf) when the value did not represent biologically feasible copy numbers. CV values were also reported in conjunction with confidence intervals and can be used to calculate standard deviations, allowing one to calculate confidence intervals when combined with mean and sample size. Student’s *t*-test with 2-way tails and unequal variances were computed on doubling time and blood sedimentation data in Microsoft Excel for Microsoft 365 for comparisons. P values were considered significant if p ≤ 0.05. Doubling time (*dt*) was assessed in hours by the following equations [38]:

$$gr=\frac{\ln\;\left(\frac{N(t)}{N(0)}\right)}{t}dt=\frac{\ln\left(2\right)}{gr}$$

where N(t) is the calculated copy number at time t, N(0) is the previous copy number, t is the time in hours since the N(0) day sample, gr is the growth rate.

## Results

### 25 cm^2^ culture growth method evaluation

**Fig 2:**
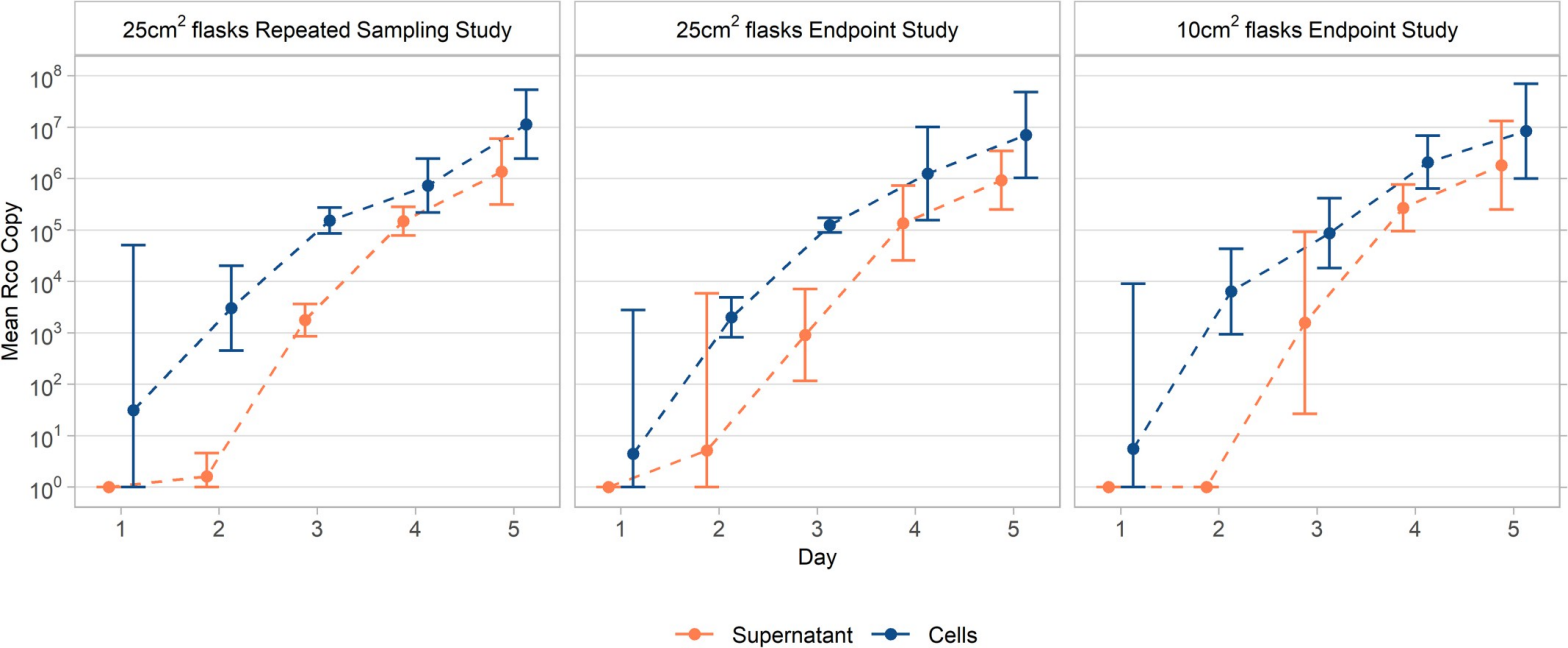
Comparison of *R*.*conorii* average total copy number in supernatant accumulation and cellular amplification with Repeated Sampling and Endpoint Methods.

RS and EP supernatant accumulation curves did not differ, as the time by study design interaction parameter was not statistically different from 0, p = 0.693, and independently, RS and EP cellular amplification curves did not differ, p = 0.324. Mean ± standard deviation of inocula were calculated to be 45.2 ± 17.7, CV = 0.392, total copies (RS) and 23.9 ± 3.0, CV = 0.127, total copies (EP). Real-time qPCR analysis revealed Rco was detectable in the 200 μL culture supernatant samples as early as 48 hours after inoculation and was consistently detected in all 3 replicates at 72 hours post inoculation, Table 1, Fig 2. Viability study results revealed viable Rco in the supernatant from day 3 to 5 post inoculation, Table 1. CPE in both the RS and EP flasks were first noted on day 4 with <1% observed CPE and peaked on day 5 with ~ 5% observed CPE, Table 1. No differences in *dt* were observed between sampling methods or in supernatant and cellular samples, S1 Table.

**Table 1:** Comparison of *R*.*conorii* in supernatant and cellular samples over time, under varying sampling and culture size conditions.

Study	Day	Sample	GM copies (95% CI)	CV	CPE (# flasks)	Viable Rco ^Δ^
**25 cm^2^ flask Repeated Sampling Study**		Inoculum	4.52x10^1^(1.14, 8.92 x10^1^) *	0.39	---	---
	1	Supernatant	0 (0, 0)	---	---	---
		Cells	3.11x10^1^ (0, 5.10 x10^4^)^B^	0.88	---	---
	2	Supernatant	1.61 (0, 4.59)^C^	3.46	---	---
		Cells	3.02x10^3^ (4.50x10^2^, 2.03x10^4^)	0.57	N	---
	3	Supernatant	1.77x10^3^ (8.61x10^2^, 3.64x10^3^)	1.05	---	---
		Cells	1.54x10^5^ (8.64 x10^4^, 2.75x10^5^)	0.24	N	---
	4	Supernatant	1.49x10^5^ (7.87x10^4^, 2.82x10^5^)	0.47	---	---
		Cells	7.37x10^5^ (2.21x10^5^, 2.46x10^6^)	0.41	Y(3)	---
	5	Supernatant	1.38x10^6^ (3.16x10^5^, 6.00x10^6^)	0.59	---	---
		Cells	1.14x10^7^ (2.44x10^6^, 5.36x10^7^)	0.49	Y(3)	---
**25 cm^2^ flask Endpoint Study**		Inoculum	2.39x10^1^ (1.63x10^1^, 3.14x10^1^) *	0.13	---	---
	1	Supernatant	0 (0, 0)	---	`---	N
		Cells	4.47 (0, 2.80x10^3^)^A^	1.73	---	---
	2	Supernatant	5.14 (0, 5.86x10^3^)^A^	1.73	---	N
		Cells	2.00x10^3^ (8.15x10^2^, 4.91x10^3^)	0.39	N	---
	3	Supernatant	9.10x10^2^ (1.16x10^2^, 7.11x10^3^)	0.87	---	Y
		Cells	1.25x10^5^ (8.99x10^4^, 1.73x10^5^)	0.13	N	---
	4	Supernatant	1.37x10^5^ (2.58x10^4^, 7.31x10^5^)	0.56	---	Y
		Cells	1.25x10^6^ (1.56x10^5^, 1.01x10^7^)	0.82	Y(3)	---
	5	Supernatant	9.30x10^5^ (2.50x10^5^, 3.46x10^6^)	0.56	---	Y
		Cells	7.12x10^6^ (1.04x10^6^, 4.86x10^7^)	0.83	Y(3)	---
**10 cm^2^ tube Endpoint Study**		Inoculum	3.74x10^1^ (2.18x10^1^, 5.30x10^1^) *	0.17	---	---
	1	Supernatant	0 (0, 0)	---	---	---
		Cells	5.58 (0, 9.09x10^3^)^A^	1.73	N	---
	2	Supernatant	0 (0, 0)	---	---	---
		Cells	6.38x10^3^ (9.38x10^2^, 4.34x10^4^)	0.74	N	---
	3	Supernatant	1.57x10^3^ (2.65x10^1^, 9.30x10^4^)	0.79	---	---
		Cells	8.72x10^4^ (1.81x10^4^, 4.19x10^5^)	0.63	Y(2)	---
	4	Supernatant	2.70x10^5^ (9.55x10^4^, 7.63x10^5^)	0.40	---	---
		Cells	2.09x10^6^ (6.42x10^5^, 6.83x10^6^)	0.42	Y(3)	---
	5	Supernatant	1.83x10^6^ (2.50x10^5^, 1.33x10^7^)	0.75	---	---
		Cells	8.41x10^6^ (1.01x10^6^, 7.04x10^7^)	0.88	Y(3)	---

Abbreviations—CI: Confidence interval; CV: Coefficient of variation; CPE: Cytopathic effects; Y: Yes; N: No

Comparison of total copies of *R*. *conorii* between 3 study designs and sample types. Geometric means of total copies and 95% confidence intervals are shown on the left, with geometric means and 95% confidence intervals on the log_10_ scale graphed on the right. *Arithmetic means and confidence intervals were used for inoculum samples. Δ denotes that viable Rco sampled from culture supernatant. All CV values >1 represent variability in data points. —, represents not assessed, A represents 1 out of 3 samples amplified, B represents 2 out of 3 samples amplified, and C represents 1 out of 12 samples amplified. Samples that were Not Detected, having zero copies were included as 0 in all calculations.

### 10 cm^2^ culture growth method evaluation

Rco supernatant accumulation curves were not statistically different when compared to 25 cm^2^ methods (time by study design interaction: RS, p = 0.263; EP, p = 0.114), and independently, cellular amplification curves were not statistically different in 10 cm^2^ culture tubes when compared to the 25 cm^2^ methods (time by study design interaction: RS, p = 0.237; EP, p = 0.208). Mean ± standard deviation of the Rco inoculum was calculated to be 37.4 ± 6.3 total copies. Copy number analysis revealed consistent detection of Rco in the culture supernatant 72 hours post inoculation at 2.8x10^3^ ± 2.2x10^3^ copies per culture tube. Similar to 25 cm^2^ flasks, cellular copy number was more concentrated than bacterial copy number in the supernatant, Table 1, Fig 2. CPE was noted as early as day 3, and by day 4 < 1% observed in all culture tubes and peaked at day 5 with ~ 2% CPE observed in all culture tubes, Table 1.

**Fig 3:**
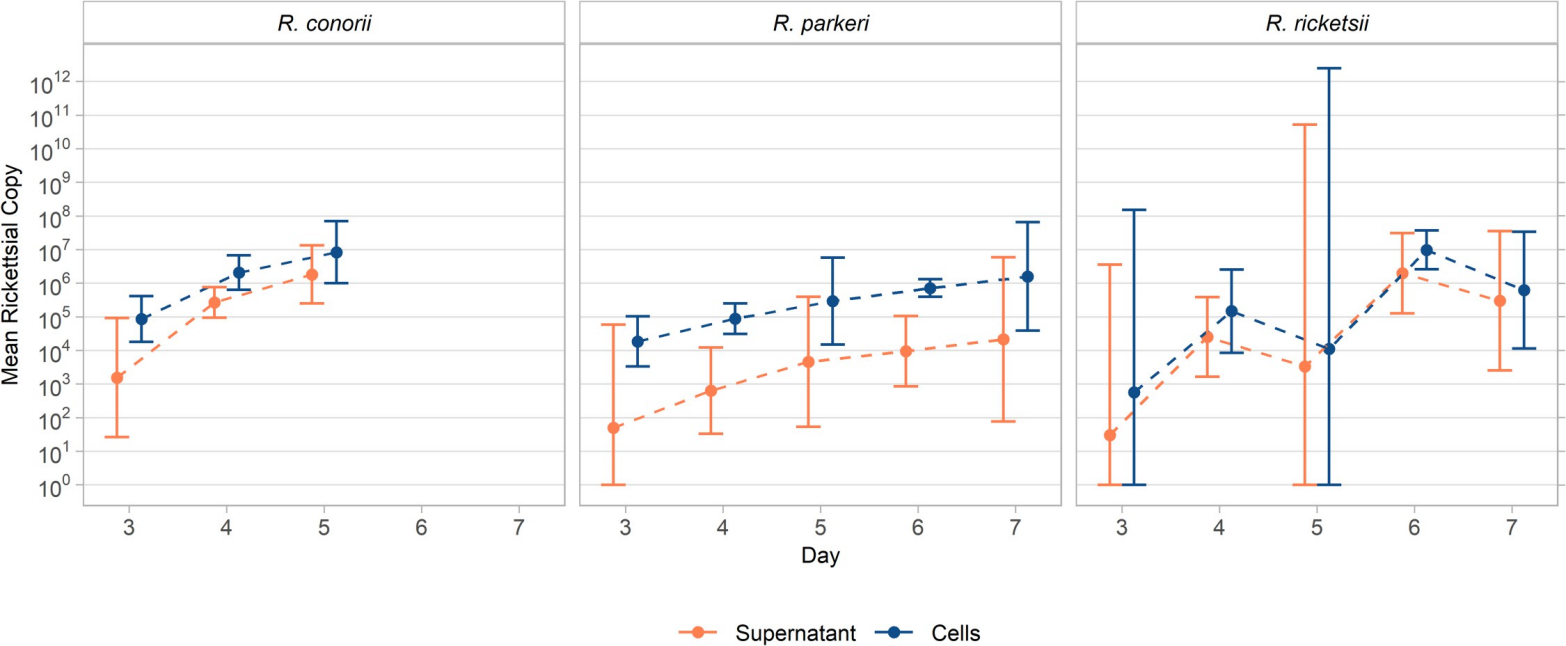
Comparison of average total copy number in supernatant accumulation and cellular amplification time course between SFGR strains.

Mean Rri and Rpa time-course inocula were calculated to be 48.3 ± 1.0 total copies of Rri or 35.7 ± 5.6 total copies of Rpa. Total copy number analysis revealed detection of both Rri (1 out of 3 samples taken) and Rpa (2 out of the 3 samples taken) 72 hours post inoculation in supernatant and cells, Table 2, Fig 3. CPE was observed sporadically throughout the time course of Rri, with plaques forming as early as day 3 in one replicate culture tube, while others had none during the time course, Table 2. Monolayer deterioration, including lifting of cells and cell lysis was noted on day 7 in Rri cultures. Accumulation of copies in the supernatant and amplification of organism in cellular samples was not statistically different across days 3 to 5 and *dt* was not statistically different across species, Table 2 and S1, Fig 3.

**Table 2:** Endpoint method comparison of average total copy number in supernatant accumulation and cellular amplification time course between SFGR strains.

Species	Day	Sample	GM copies (95% CI)	CV	CPE (# flasks)
***R*. *conorii***		Inoculum	3.74x10^1^ (2.18x10^1^, 5.30x10^1^) *	0.168	---
	Day 3	Supernatant	1.57x10^3^ (2.67x10^1^, 9.26x10^4^)	0.794	---
		Cells	8.72x10^4^ (1.81x10^4^, 4.19x10^5^)	0.634	Y (2)
	Day 4	Supernatant	2.70x10^5^ (9.55x10^4^, 7.63x10^5^)	0.397	---
		Cells	2.09x10^6^ (6.42x10^5^, 6.83x10^6^)	0.424	Y (3)
	Day 5	Supernatant	1.83x10^6^ (2.50x10^5^, 1.33x10^7^)	0.747	---
		Cells	8.41x10^6^ (1.01x10^6^, 7.04x10^7^)	0.879	Y (3)
***R*. *parkeri***		Inoculum	3.57x10^1^ (2.17x10^1^, 4.97x10^1^) *	0.158	---
	Day 3	Supernatant	4.99x10^1^ (4.16x10^-2^, 5.97x10^4^)	0.716	---
		Cells	1.86x10^4^ (3.37x10^3^, 1.03x10^5^)	0.725	N
	Day 4	Supernatant	6.45x10^2^ (3.34x10^1^, 1.24x10^4^)	0.431	---
		Cells	8.81x10^4^ (3.10x10^4^, 2.50x10^5^)	1.432	N
	Day 5	Supernatant	4.61x10^3^ (5.33x10^1^, 3.99x10^5^)	0.828	---
		Cells	2.97x10^5^ (1.50x10^4^, 5.86x10^6^)	0.975	N
	Day 6	Supernatant	9.54x10^3^ (8.56x10^2^, 1.06x10^5^)	0.254	---
		Cells	7.23x10^5^ (3.97x10^5^, 1.32x10^6^)	1.309	N
	Day 7	Supernatant	2.15x10^4^ (7.72x10^1^, 5.98x10^6^)	1.332	---
		Cells	1.60x10^6^ (3.89x10^4^, 6.55x10^7^)	1.194	N
***R*. *rickettsii***		Inoculum	4.83x10^1^ (4.57x10^1^, 5.08x10^1^) *	0.021	---
	Day 3	Supernatant	3.03x10^1^ (2.52x10^-4^, 3.64x10^6^)^A^	1.731	---
		Cells	5.71x10^2^ (2.10x10^-3^, 1.55x10^8^)^B^	1.513	Y (1)
	Day 4	Supernatant	2.56x10^4^ (1.68x10^3^, 3.91x10^5^)	1.097	---
		Cells	1.49x10^5^ (8.63x10^3^, 2.57x10^6^)	1.109	Y (2)
	Day 5	Supernatant	3.37x10^3^ (2.14x10^-4^, 5.30x10^10^)^B^	1.658	---
		Cells	1.11x10^4^ (4.97 x10^-5^, 2.49 x10^12^) ^B^	1.688	Y (1)
	Day 6	Supernatant	1.99x10^6^ (1.27x10^5^, 3.14x10^7^)	0.806	---
		Cells	9.85x10^6^ (2.61x10^6^, 3.71x10^7^)	0.439	Y (2)
	Day 7	Supernatant	3.01x10^5^ (2.55x10^3^, 3.54x10^7^)	1.513	---
		Cells	6.28x10^5^ (1.16x10^4^, 3.41x10^7^)	1.387	Y (1)

Abbreviations—CI: Confidence interval; CV: Coefficient of variation; CPE: Cytopathic effects; Y: Yes; N: No

Comparison of total copies between study designs and sample types across 3 rickettsial species. Geometric means of total copies and 95% confidence intervals are shown on the left, with geometric means and 95% confidence intervals on the log_10_ scale graphed on the right.

*Arithmetic means and confidence intervals were used for inoculum samples. All CV values >1 represent variability in data points. —, indicates Not Accessed, A, indicates 1 out of 3 sampled from individual flasks amplified, B, indicates 2 out of 3 samples amplified. CPE observed, Y (yes) or N (no) followed by the number out of 3 flasks for each time point CPE was present.

### Whole blood sedimentation

**Fig 4:**
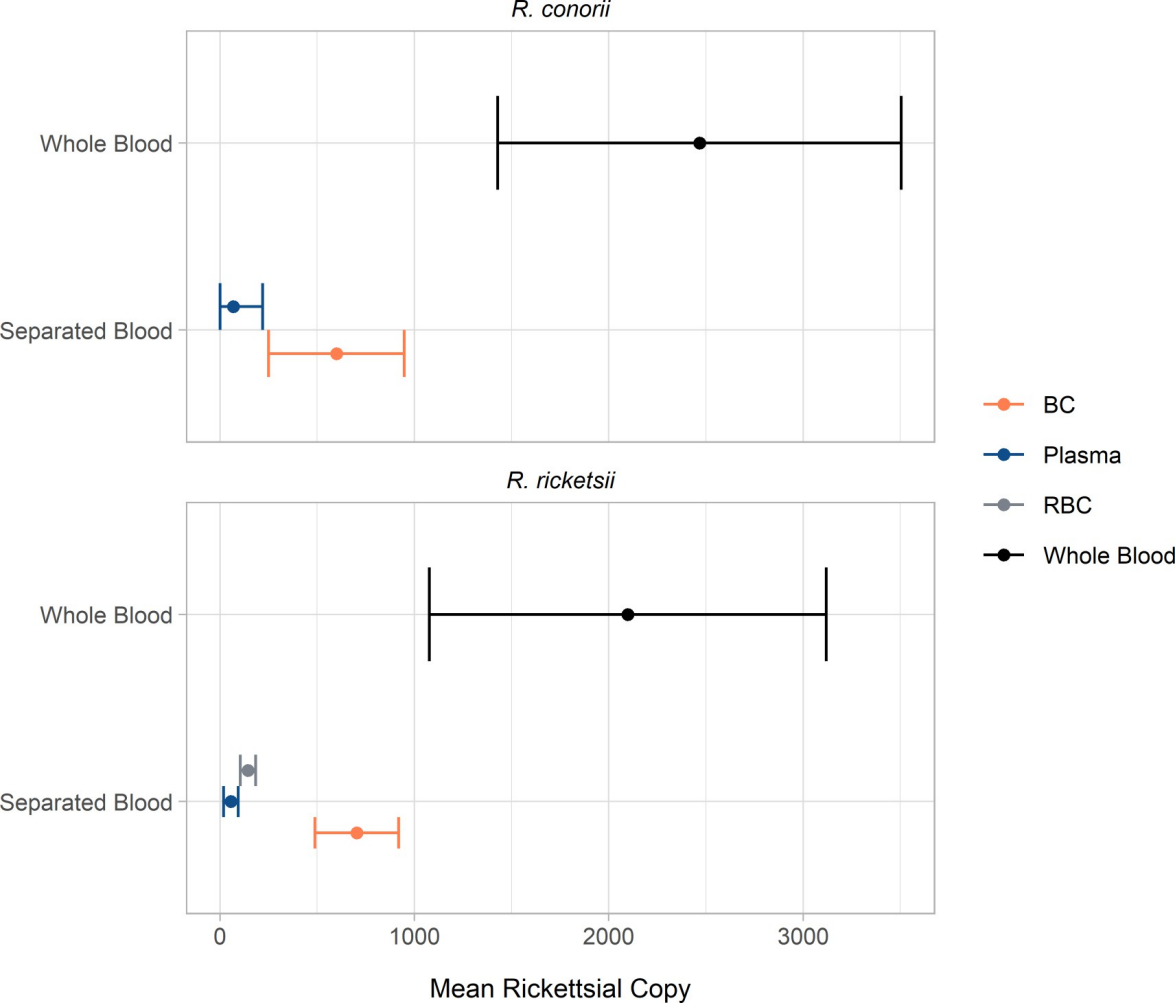
Post-Sedimentation effects on rickettsial copy number recovery. Rco and Rri whole blood separation. Whole blood was inoculated with approximately 1,800 copies/mL of either Rri or Rco and split into 3 (Rco) or 6 (Rri) 1 mL aliquots sedimented with Histopaque 1077 gradient. Entire separated blood layers including plasma, BC and RBC (Rri only) were taken for quantification by qPCR. Data represents the averaged total copy number from each blood layer. Whole blood total copy number was averaged from triplicate aliquots of unseparated inoculated whole blood and calculated to 1 mL. All samples were analyzed by PanR8 qPCR in duplicate.

WB inoculated with Rri or Rco showed a loss of rickettsial copies when fractionated by centrifugation and analyzed by fraction (plasma, BC, and RBC layers). Blood component copy number was determined by the PanR8 qPCR to be 55.7 ± 36.1 copies and 137.4 ± 121.2 copies for plasma, 703.7 ± 205.1 copies and 754.5 ± 259.1 for BC for Rri and Rco, respectively. Rri RBC were 144.1 ± 37.7 copies. Whole blood copy number analysis was reported as 2097.9 ± 973.1 copies and 2467.2 ± 417.6 copies for Rri and Rco, respectively. Compared to the WB for Rri and Rco recovery, respectively, the total of the sedimented blood phases had a recovery of 43% (Rri) [p = 0.03] and 27% (Rco) [p = 0.01], Fig 4. RBC for Rco were not analyzed due to an extraction failure caused by sample viscosity leading to sample loss from overwhelmed magnetic bead capacity of the MagNa Pure extraction unit. Rri RBC fractions were diluted prior to extraction to overcome the viscosity issue.

### Clinical sample application

Of the 25 samples from 19 patients, 10 were positive for rickettsial DNA via the PanR8 qPCR assay (C001, C004, C006, C011, C012, C015, C020, C028, and C029), and of those, 7 isolates of *R*. *rickettsii* were cultivated from samples C001, C004, C011, C012, C015, C020, and C029. Of the 18 unsuccessful isolation attempts, 4 were contaminated with an unknown organism (C006 and C008, drawn post-mortem collection and 4 days post-doxycycline administration; C013, drawn pre-doxycycline administration with no detectable rickettsial DNA; and C027, drawn pre-doxycycline administration and was not tested at the time of receipt as it did not meet the diagnostic sample testing criteria), 5 were drawn 1–5 days post-doxycycline administration (C010, C014, C016, C024, and C026 with C024 drawn 1 day post-doxycycline administration and was qPCR positive for rickettsial DNA), 8 were drawn pre-doxycycline administration with no detectable rickettsial DNA in the original sample (C002, C003, C005, C017, C019, C021, C023, and C025), and 1 drawn pre-doxycycline administration (C008) did not meet testing criteria and was not tested at the time of receipt.

Isolates were derived from WB, plasma and serum samples drawn before or at the time of doxycycline administration, and within 5 days of symptom onset, Table 3. Of the 7 isolates obtained, C001, Rri La Crosse, had detectable logarithmic accumulation of Rri in the supernatant as early as 48 hours post inoculation, Rri AZ C020 and Rri AZ C029 had detectable logarithmic accumulation of Rri in the supernatant at 7 days post inoculation, while Rri VA C004, Rri AZ C011, Rri AZ C012 and Rri AZ C015 isolates only had logarithmic accumulation of Rri in the supernatant after 1 passage into a 25 cm^2^ flask, Table 4. No antibiotics were used in the culture media, and as a result 4 samples were unable to be cultured due to contamination. The contaminated sample cultures did not have detectable rickettsial DNA via the PanR8 real-time PCR. Samples C002 and C003, from a confirmed RMSF patient, had no detectable rickettsial bacteria upon initial testing were found to be positive post-culture attempt as the specimen was diluted in culture medium. Isolate *dt* was assessed during logarithmic growth at either passage 0 or passage 1 and ranged between 5.5 ± 2.3 hr and 16.4 ± 6.3 hr, S2 Table.

**Table 3:** Summary of clinical samples.

Sample Identifier	Patient No.	State	Specimen Type	Fatality	Number of Days sample Drawn from Onset	Doxycycline Administration		Original Sample PanR8 Result, Averaged CT Value	Culture Supernatant PanR8 Result from Day 7–11 passage 0	Isolation Result
						Pre/Post Sample Draw	Days from Administration to Draw			
C005	1	MT	serum	N	1	Pre	0	Not Detected	Not Detected	No Growth
C002	2	VA	serum	Y	2	Pre	-1	Not Detected^Δ^	Positive^Δ^	No Growth
C003	2	VA	serum	Y	2	Pre	-1	Not Detected^Δ^	Positive^Δ^	No Growth
C004	2	VA	WB	Y	0	Pre	-3	Positive, 33.57	Positive	*R*. *Rickettsii* VA C004
C006*	2	VA	WB	Y^Ψ^	7	Post	4	Positive, 31.95	Not Detected	Contaminated
C008*	2	VA	WB	Y^Ψ^	7	Post	4	Not tested	Not Detected	Contaminated
C010	4	AZ	WB	Unknown	11	Post	3	Not Detected	Not Detected	No Growth
C011	5	AZ	plasma	Y	1	N/A	N/A	Positive, 26.95	Positive	*R*. *Rickettsii* AZ C011
C012	5	AZ	serum	Y	1	N/A	N/A	Positive, 26.40	Positive	*R*. *Rickettsii* AZ C012
C013	6	WI	WB	Y	3	Pre	0	Not Detected	Not Detected	Contaminated
C014	6	WI	WB	Y^Ψ^	8	Post	5	Not Detected	Not Detected	No Growth
C017	7	UT	WB	Unknown	1	Pre	0	Not Detected	Not Detected	No Growth
C016	8	MA	WB	N	12	Post	1	Not Detected	Not Detected	No Growth
C001	9	WI	WB	Y	0	Pre	0	Positive, 30.16	Positive	*R*. *Rickettsii* La Crosse
C015	10	AZ	WB	Y	1	Pre	0	Positive, 27.78	Positive	*R*. *Rickettsii* AZ C015
C019	19	CO	serum	Unknown	19	Pre	-2	Not Detected	Not Detected	No Growth
C020	20	AZ	WB	Y	4	Pre	0	Positive, 31.80	Positive	*R*. *Rickettsii* AZ C020
C021	21	SC	serum	N	0	Pre	0	Not Detected	Not Detected	No Growth
C023	23	UT	serum	N	1	Pre	0	Not Detected	Not Detected	No Growth
C024	29	WA	serum	N	8	Post	1	Positive, 36.20	Not Detected	No Growth
C025	31	MI	serum	N	5	Pre	0	Not Detected	Not Detected	No Growth
C026	32	MA	serum	N	11	Post	1	Not Detected	Not Detected	No Growth
C027	33	AZ	WB	Unknown	6	Pre	1	Not tested	Not Detected	Contaminated
C028	34	AZ	WB	Y	0	Pre	0	Positive, 29.28	Positive	No Growth
C029	35	AZ	WB	N	5	Pre	-1	Positive, 30.42	Positive	*R*. *Rickettsii* AZ C029

Summary of clinical sample data used for isolation.

* indicates duplicate aliquots from the same original sample collection tube were tested separately with same result, Δ indicates PCR inhibition in original sample was overcome by dilution into culture media, WB indicates whole blood. Ψ indicated sample was drawn post-mortem.

**Table 4:** Isolate growth time course.

	*R*. *Rickettsii* VA C004			*R*. *Rickettsii* AZ C011			*R*. *Rickettsii* AZ C012			*R*. *Rickettsii* La Crosse			*R*. *Rickettsii* AZ C015			*R*. *Rickettsii* AZ C020			*R*. *Rickettsii* AZ C029		
Day / Passage	GM (95% CI)	CV	CPE (%)	GM (95% CI)	CV	CPE (%)	GM (95% CI)	CV	CPE (%)	GM (95% CI)	CV	CPE (%)	GM (95% CI)	CV	CPE (%)	GM (95% CI)	CV	CPE (%)	GM (95% CI)	CV	CPE (%)
**2/0**	**2.44x10^1^** (0, Inf)	1.414	0	**6.13x10^3^** (1.95x10^1^, 1.93x10^6^)	0.600	0	**4.27x10^3^** (4.51x10^2^, 4.04x10^4^)	0.248	0	**6.34x10^1^** (0, Inf)	1.414	0	**5.13x10^3^** (2.07x10^3^, 1.27x10^4^)	0.101	0	0 (0, 0)	---	0	0 (0, 0)	---	0
**3/0**	**3.10x10^1^** (0, Inf)	1.414	0	**4.92x10^3^** (2.95x10^1^, 8.22x10^5^)	0.541	0	**2.34x10^3^** (7.45x10^1^, 7.33x10^4^)	0.375	0	**1.11x10^4^** (3.26x10^2^, 3.79x10^5^)	0.383	0	**4.96x10^4^** (2.83x10^4^, 8.68x10^4^)	0.062	0	NS	NS	-	NS	NS	-
**4/0**	**2.31x10^1^** (0, Inf)	1.414	0	**6.28x10^3^** (3.30x10^3^, 1.19x10^4^)	0.071	0	**2.42x10^3^** (9.94, 5.88x10^5^)	0.576	0	**5.50x10^4^** (1.37x10^4^, 2.21x10^5^)	0.154	1	**1.31x10^5^** (9.31x10^4^, 1.85x10^5^)	0.038	1	**9.50x10^3^** (4.24x10^2^, 2.13x10^5^)	0.339	0	**1.06x10^5^** (8.33x10^4^, 1.35x10^5^)	0.167	0
**5/0**	0	---	0	**7.17x10^3^** (3.71x10^3^, 1.38x10^4^)	0.073	0	**2.89x10^3^** (6.77x10^1^, 1.23x10^5^)	0.406	0	**5.11x10^5^** (4.66x10^5^, 5.60x10^5^)	0.010	2	**2.24x10^5^** (1.38x10^5^, 3.63x10^5^)	0.054	1	NS	NS	-	NS	NS	-
**6/0**	0	---	0	NS	NS	-	NS	NS	-	**2.58x10^6^** (2.47x10^6^, 2.70x10^6^)	0.005	10	NS	NS	-	NS	NS	-	NS	NS	-
**7/0**	**9.39x10^2^** (7.74x10^1^, 1.14x10^4^)	0.275	0	**2.21x10^3^** (0, Inf)	1.214	0	**3.88x10^3^** (3.15x10^3^, 4.79x10^3^)	0.023	0	**7.56x10^6^** (5.03x10^6^, 1.14x10^7^)	0.045	10	**2.83x10^5^** (2.62x10^5^, 3.07x10^5^)	0.009	0	**5.31x10^5^** (1.82x10^5^, 1.55x10^6^)	0.119	0	**8.05x10^6^** (7.74x10^6^, 8.37x10^6^)	0.004	10
**8/0**	NC	NC	NC	NS	NS	-	NS	NS	-	NC	NC	NC	NS	NS	-	**8.20x10^5^** (7.60x10^5^, 8.84x10^5^)	0.008	0	**1.33x10^7^** (1.11x10^7^, 1.58x10^7^)	0.020	20
**9/0**	NC	NC	NC	NS	NS	-	NS	NS	-	NC	NC	NC	NS	NS	-	**1.07x10^6^** (9.08x10^5^, 1.27x10^6^)	0.019	0	NC	NC	NC
**10/0**	NC	NC	NC	NS	NS	-	NS	NS	-	NC	NC	NC	NS	NS	-	**2.54x10^6^** (6.71x10^5^, 9.62x10^6^)	0.148	0	NC	NC	NC
**11/0**	NC	NC	NC	**4.97x10^3^** (2.14x10^2^, 1.15x10^5^)	0.343	-	**7.11x10^3^** (6.82x10^3^, 7.41x10^3^)	0.005	-	NC	NC	NC	**9.56x10^5^** (4.95x10^5^, 1.85x10^6^)	0.073	-	**3.34x10^6^** (9.73x10^5^, 1.15x10^7^)	0.137	2^B^	NC	NC	NC
**1/1**	**3.81x10^1^** (0, Inf)	1.414	0	**4.42x10^1^** (0, Inf)	1.414	0	NS	NS	0	NC	NC	NC	**1.15x10^5^** (3.22x10^4^, 4.11x10^5^)	0.141	0	NC	NC	NC	NC	NC	NC
**2/1**	**2.17x10^3^** (2.03x10^3^, 2.33x10^3^)	0.008	0	**4.14x10^1^** (0, Inf)	1.414	0	NS	NS	0	NC	NC	NC	**5.35x10^5^** (4.84x10^5^, 5.92x10^5^)	0.011	0	NC	NC	NC	NC	NC	NC
**3/1**	**3.06x10^4^** (2.24x10^4^, 4.19x10^4^)	0.035	0	**3.75x10^4^** (3.19x10^3^, 4.42x10^5^)	0.271	1	**4.00x10^1^** (0, Inf)	1.414	0	NC	NC	NC	**9.93x10^6^** (5.48x10^6^, 1.80x10^7^)	0.066	5	NC	NC	NC	NC	NC	NC
**4/1**	**7.33x10^4^** (3.71x10^4^, 1.45x10^5^)	0.076	0	**3.21x10^5^** (6.16x10^4^, 1.67x10^6^)	0.183	5	**4.05x10^1^** (0, Inf)	1.414	0	NC	NC	NC	**5.92x10^7^** (3.98x10^6^, 8.81x10^8^)	0.296	20	NC	NC	NC	NC	NC	NC
**7/1**	**1.95x10^5^** (1.14x10^5^, 3.33x10^5^)	0.060	1	**3.07x10^7^** (1.25x10^7^, 7.56x10^7^)	0.100	10	**4.50x10^3^** (3.61x10^1^, 5.62x10^5^)	0.513	5	NC	NC	NC	NC	NC	NC	NC	NC	NC	NC	NC	NC
**9/1**	**3.91x10^5^** (6.58x10^4^, 2.32x10^6^)	0.197	0^A^	**5.84x10^7^** (1.38x10^6^, 2.47x10^9^)	0.405	20–30	**2.70x10^4^** (9.90x10^3^, 7.34x10^4^)	0.111	5^A^	NC	NC	NC	NC	NC	NC	NC	NC	NC	NC	NC	NC

Clinical isolates which have copy number amplification within the first 7 days of culture are La Crosse, AZ C020, and AZ C029. Clinical isolates which have copy number amplification after the first passage of culture are VA C004, AZ C011, AZ C012, and AZ C015. Geometric means (GM) of total copies and 95% confidence intervals of duplicate real-time PCR reactions for clinical samples from 7 primary isolates of *R*. *rickettsii*. RS method used with 1, 10 cm^2^ culture tube per sample, 1 supernatant sample taken per time point. All CV values >1 represent high variability in data points.

^A^, indicates culture was monitored to day 14–18 until at least 20% CPE was observed and *Rickettsia* was visualized via stain, ND, indicates Not Detected, NS, indicates no sample taken, NC, indicates not cultured to this time point, Inf, indicates calculated confidence interval was not biologically feasible, and—indicates no monitoring occurred.

## Discussion

**Fig 5:**
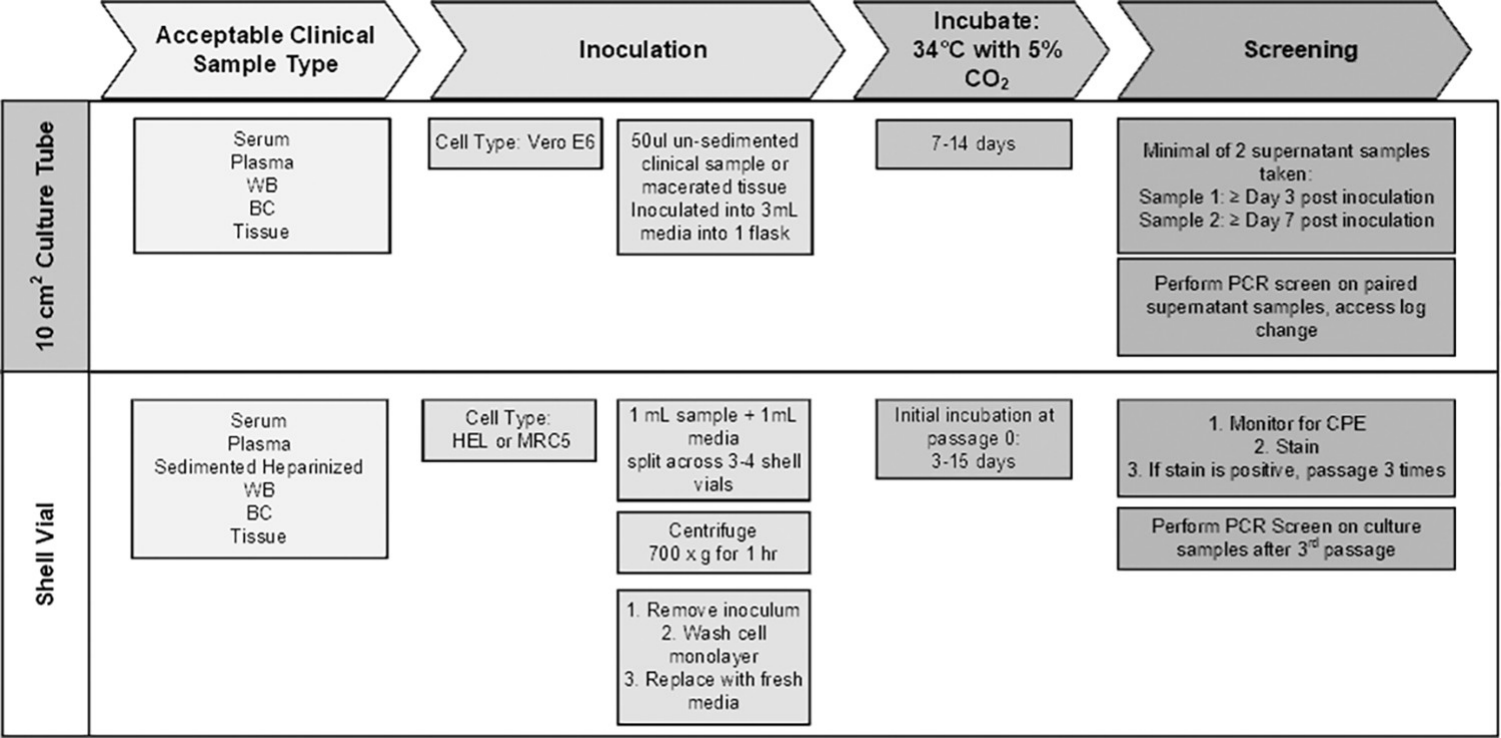
Clinical Isolation Method Comparison.

Minimal improvements have been made on the shell vial technique for clinical sample diagnostics since inception. We show that diagnostic SFGR culture can be more versatile and be used with less manual manipulation than traditionally required. Our refinements provide a safe procedure by using a robust cell type, changing the culture apparatus, culture volume, and primary detection method. The limitations of shell vial culture include the use of multiple vials per patient sample [26], specimen type, and the methods for monitoring the culture over time. There are also safety considerations with the vial closure. Specimens in EDTA, a common blood collection tube additive used for SFGR diagnostics, are restricted from shell vial procedures due to disruption to HEL or MRC5 cell monolayers and interference with staining [22,29,39]. The use of Vero E6 cells in shell vial has been noted in non-human research capacities [40], however, using this cell type in a diagnostic application has not been previously described. Whole blood containing RBCs causes background in staining methods [22,29], requiring RBC sedimentation and removal prior to inoculation. Furthermore, additional handling for staining and visual monitoring of cultures requires specialized training and will vary between technicians and poses increased safety risk. We provide a simplified method for culture as outlined in Fig 5 utilizing one 10 cm^2^ culture tube per clinical sample, and such tube has a wide mouth opening for easy manipulation to sample the supernatant and a hydrophobic filtered screw top lid for safe movement outside of a biological safety cabinet. This safety feature is critical as accumulation of viable Rco in the supernatant is seen 3 days post inoculation, Table 1, Fig 2. The increased media volume of 3 mL sustains the culture for up to 14 days, allowing for repeated supernatant sampling. This volume also dilutes the small volume clinical material inoculum allowing for unimpeded visual monitoring.

Similar doubling times were observed between sampling methods, suggesting that SFGR accumulate at the same rate in cells and supernatant regardless of culture apparatus, S1 Table. This demonstrates that supernatant sampling is an appropriate method to assess early growth dynamics [23,41] and reflects agent replication. Of note, trends in media copy number accumulation mirror those of cells on a one day delay, Table 1, Fig 2, suggesting that copy number values in supernatant could project that of cells the day before, however, more research must be done to expand this idea. Due to its utility and convenience, the EP method was applied to the 10 cm^2^ culture tube validation in Rco, Rri, and Rpa, and it showed reduced cellular surface area had consistent early detection equivalent to 25 cm^2^ flasks and shell vial [10] for Rco. Further validation was performed on low passage isolates *R*. *rickettsii* AZ3 strain and *R*. *parkerii* Coweta strain, as lag phase growth during initial isolation varies between SFGR species and are dependent on the culture system and amount of viable bacterium in the inoculum [42–44]. Differences in copy number accumulation, *dt*, and observed CPE between strains was seen, however, all were detected in the media as early as day 3, and consistently by day 4, Table 2.

An evaluation to understand the effects of the common practice of WB sedimentation by centrifugation was performed by Histopaque 1077 gradient prior to isolation [29] and determined a loss in copy number recovery per blood phase of contrived WB infected samples of 57% (Rri) and 73% (Rco), Fig 4. It remains unknown if this is partly due to an inefficient extraction process or is due to PCR inhibition from the Histopaque 1077 gradient. Sedimentation and buffy coat retrieval without Histopaque treatment should also be assessed. RBC were not analyzed for Rco contrived samples, as based on the Rri data the total copies recovered from RBC was 16% and accounts for the disparity on recovery between species. Further studies must be done to determine the cause of this loss, however, a method without sample sedimentation ensures maximum isolation efficiency.

Validation was performed with 25 clinical specimens, of which 16 specimens met the previously established criteria [13,22,32] for culture isolation of being drawn before doxycycline administration; two did not have antibiotic treatment data available. Small volumes of 50 μL of original clinical WB, serum, or plasma frozen with 50 μL of SPG or 50 μL fresh specimen mixed 50 μL of SPG, were inoculated directly into culture for a total volume of 3 mL. A combination of dilution factor of the whole blood inoculum with the use of the Vero E6 cells resulted in no interference with monolayer visualization for all but 1 sample (C016, fresh WB) and no cellular lysis due to RBC. Further evaluation must be done to determine differences in the use of fresh over frozen samples. In this group of samples, successful isolations were obtained from blood, serum, and plasma specimens drawn within 5 days of onset, before or at the time of doxycycline administration, and were originally positive with the PanR8 real-time PCR assay, Table 3, confirming parameters previously determined for optimal isolation [13,22,32]. Of the 16 samples appropriate for culture, 6 were positive with the PanR8 real-time PCR assay, and of those 6 positive samples, 5 established isolates (83%). The 2 samples that did not have antibiotic treatment data available did establish isolates and were positive with the PanR8 real-time PCR assay but were not included in this metric.

In this model, no media changes occur, and original specimen material is not removed. Therefore, if copies of rickettsial DNA exist in a sample, they persist in the supernatant and are detectable throughout the time course. Only 3 out of 7 isolates showed logarithmic copy number increase within the first 7 days of culture. Clinical isolations were performed in rounds of 5–6 attempts, where the first 2 rounds were only taken to day 11 and frozen back, resulting in the need to passage to confirm if positive qPCR results showed viable organism or residual bacterial DNA from the inoculum. This resulted in the detection of 4 additional isolates, Table 4. For the remaining attempts, all cultures were monitored to day 18–22, or until CPE was observed without media change (unless contaminated), at which time cultures were sampled to confirm no growth via staining. Deterioration of the Vero E6 monolayer was not noted until day 14 of culture. These data show that as a practical application for a clinical isolation model, at least an early (day 3–6) and late (day 7–14) timepoint sample must be taken to determine log increases in copy number to confirm growth if CPE is not observed. This is required in order to overcome residual inoculum copy number that may confound results. To use this model in a research capacity, multiple samples can be taken over the 14 day period, however, the number of samples taken must be taken into consideration to ensure a minimal media coverage by day 14.

This investigation provides justification for a small volume model for clinical rickettsial isolation that can be used with limited sample volume. Unlike previously established techniques, this model allows for the use of unprocessed WB and eliminates post-inoculation centrifugation and media change. Importantly, this model requires only a small amount of clinical sample inoculum, allowing for culture attempts from limited volume samples, defines a culture window from which sampling can occur to limit manipulation, and focuses on confirmation of growth via qPCR rather than observation and staining. Repeated sampling can provide valuable isolate growth data at early time points and isolate passage, and in a diagnostic setting, two samples, early and late in culture, may be enough to confirm growth before the appearance of CPE. Assuming that a sample contains viable SFGR at the time of receipt, this model provides a safe and effective method for isolation from limited clinical material.

## Supporting information

S1 Table Summary of supernatant and cellular doubling time (*dt*). The averaged data is represented as average *dt* ± standard deviation. Rco *dt* reported as starting on day 2 for T25 supernatant, however consistent sampling occurred on day 3.
(DOCX)

S2 Table Summary of isolate doubling time (*dt*). Data represented as average dt ± standard deviation. Days of log growth at p1 indicated *dt* was determined by data from growth confirmation in 25 cm^2^ culture flask at isolate passage 1.
(DOCX)

S1 Data Excel spreadsheet containing, in separate sheets, the underlying numerical data and statistical analysis for Tables 1, 2, 4, S1 and S2 and Figs 2, 3 and 4. (XLSX)
