# Supplementary material for: Procedure for spotted fever group Rickettsia isolation from limited clinical blood specimens
Source: PLoS Negl Trop Dis. 2022 Oct 14;16(10):e0010781. doi: 10.1371/journal.pntd.0010781 (PMC9605293; doi:10.1371/journal.pntd.0010781)
Supplement: S1 Table — The averaged data is represented as average dt ± standard deviation. Rco dt reported as starting on day 2 for T25 supernatant, however consistent sampling occurred on day 3. (DOCX) [file pntd.0010781.s001.docx]

**S1 Table.** Summary of supernatant and cellular doubling time (dt).

| **Study** |  | **dt (hr)** | **Days of Log Growth** |
| --- | --- | --- | --- |
| **Rco RS Study 25cm^2^ flasks** | **Supernatant** | 5.17 ± 2.39 | 2-5 |
|  | **Cells** | 6.39 ± 2.51 | 1-5 |
| **Rco EP study 25cm^2^ flasks** | **Supernatant** | 5.77 ± 2.74 | 2-5 |
|  | **Cells** | 6.35 ± 3.06 | 1-5 |
| **Rco 10 cm^2^ culture tubes** | **Supernatant** | 7.59 ± 3.79 | 2-5 |
|  | **Cells** | 6.37 ± 3.25 | 1-5 |
| **Rri in 10 cm^2^ culture tubes** | **Supernatant** | 8.39 ± 2.49 | 0-6 |
|  | **Cells** | 7.99 ± 2.09 | 0-6 |
| **Rpa in 10 cm^2^ culture tubes** | **Supernatant** | 13.91 ± 8.31 | 0-7 |
|  | **Cells** | 10.47 ± 1.87 | 0-7 |
