# Supplementary material for: Procedure for spotted fever group Rickettsia isolation from limited clinical blood specimens
Source: PLoS Negl Trop Dis. 2022 Oct 14;16(10):e0010781. doi: 10.1371/journal.pntd.0010781 (PMC9605293; doi:10.1371/journal.pntd.0010781)
Supplement: S2 Table — Data represented as average dt ± standard deviation. Days of log growth at p1 indicated dt was determined by data from growth confirmation in 25 cm2 culture flask at isolate passage 1. (DOCX) [file pntd.0010781.s002.docx]

**S2 Table.** Summary of isolate doubling time (dt)

| **Isolate** | **dt (hr)** | **Days of Log Growth** |
| --- | --- | --- |
| ***R. Rickettsii* VA C004** | 16.4 ± 6.3 | 1-9 p1 |
| ***R. Rickettsii* AZ C011** | 5.5 ± 2.3 | 2-7 p1 |
| ***R. Rickettsii* La Crosse** | 10.7 ± 2.6 | 2-7 |
| ***R. Rickettsii* AZ C015** | 8.7 ± 2.7 | 1-4 p1 |
| ***R. Rickettsii AZ C020*** | 12.6 | 4-7 |
| ***R. Rickettsii AZ C029*** | 11.4 | 4-7 |
